# A Case of Implantable Collamer Lens (ICL) with Reverse Orientation for 10 Years

**DOI:** 10.1155/2021/6641475

**Published:** 2021-04-14

**Authors:** Bader S. AlQahtani, Abdulaziz A. Alshamrani, Raed E. Alsulami, Jose M. Vargas

**Affiliations:** ^1^King Abdulaziz Medical City, Jeddah, Saudi Arabia; ^2^King Khaled Eye Specialist Hospital, Riyadh, Saudi Arabia; ^3^Jeddah Eye Hospital, Jeddah, Saudi Arabia

## Abstract

**Background:**

Implantable collamer lens (ICL) might rarely cause complications that warrant explantation. Here, we presented a case of inverted ICL that has undergone explantation after a 10-year duration. *Case Presentation*. A 49-year-old male with past ocular history of ICL implantation 10 years ago presented complaining of progressive decrease of visual acuity in the left eye for a long time. On examination, decreased visual acuity, anterior subcapsular cataract, and low ICL vault were noted. The anterior segment optical coherence tomography (AS OCT) showed inverted ICL. The ICL was explanted, and cataract surgery was done.

**Conclusion:**

Inverted ICL should be kept in mind as a rare cause of long-term poor visual acuity in patient with a history of ICL surgery.

## 1. Background

The advent of phakic intraocular lens (pIOL) has made a breakthrough alternative in the management of myopic patients who are unfit for laser vision correction. Currently, the only FDA-approved posterior chamber pIOL is Visian implantable collamer lens (ICL, STAAR Surgical Co., Monrovia, CA). Although it showed comparable outcomes in terms of safety, efficacy, predictability, and stability, some patients might experience complications that warrant explantation. A study was done by Alsabaani et al. where causes of ICL explantation were reviewed and analyzed [1]. It was found that ICL explantation rate was less than 4% and improper sizing with poor vaulting showed to be the main cause of explantation. They reported maximum duration between implantation and explantation to be 30 months. Here, we presented a case of inverted ICL that has undergone explantation after a duration of 10 years.

## 2. Case Presentation

**Figure 1:**
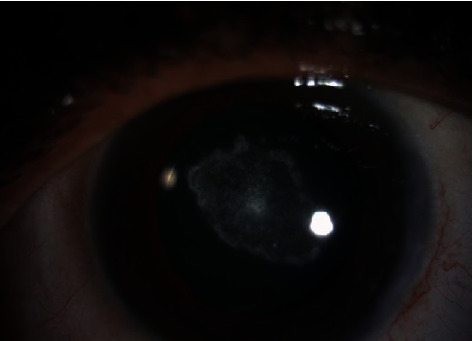
Preoperative anterior segment photo of the left eye.

**Figure 2:**
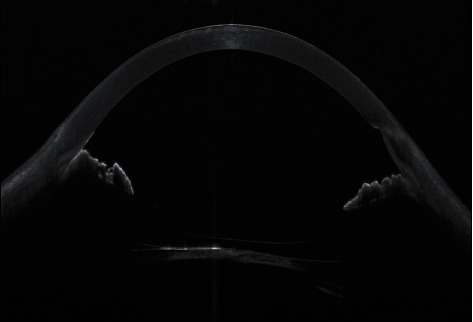
AS OCT of the left eye.

A 49-year-old male presented complaining of progressive decrease of the visual acuity in the left eye for a long time. His past ocular history includes keratorefractive surgery in both eyes 25 years ago and ICL implantation in the left eye 10 years ago by another ophthalmologist. He has also been treated for unilateral glaucoma in the left eye with dorzolamide/timolol drops twice a day. On examination, the unaided visual acuity was 20/25 OD and 20/160 OS and the intraocular pressure was 15 OD and 19 OS. The slit lamp examination of the right eye was within normal limit apart from early nuclear cataractous changes while the examination of the left eye showed the ICL in sulcus with very low vault size and 2+ anterior subcapsular cataract Figure 1. The fundus exam was normal in both eyes. Further investigations were requested including AS OCT which showed inverted ICL Figure 2. After discussion with the patient, we decided to proceed for ICL explantation with cataract surgery and IOL insertion of the left eye. Explantation surgery was done, and the reverse orientation was confirmed intraoperatively by observing the ICL haptic marks which were visible on the leading left haptic and the trailing right haptic. The patient was seen postoperatively with unaided visual acuity of 20/30 OS and with controlled IOP on antiglaucoma drop.

## 3. Discussion and Conclusion

The ICL was approved by the FDA in 2005 as an alternative for patients with moderate to high degrees of myopia [2]. It showed to be an effective, safe, and reversible refractive procedure.

Moreover, the rate of ICL complications reported was very low [1]. Some of these complications can be managed conservatively with medical treatment and close observation while others might warrant surgical intervention including ICL change or explantation.

The rate of ICL exchange/explantation was reported to be between 1% and 3.8% [1, 2]. Reasons for that include cataract formation, retinal detachment, improper sizing, high residual astigmatism, glare, high intraocular pressure, and recurrent uveitis [1, 3]. Inverted/reverse orientation of the ICL is reported as a rare complication that mostly leads to low vault and subsequently anterior subcapsular cataract formation [3]. The reverse orientation can be confirmed intraoperatively by looking for the flipped ICL haptic marks. In normally oriented ICL, there are square etchings on the corners of the ICL haptics. They should be on the leading right haptic and the trailing left haptic to confirm the correct position intraoperatively [4].

Explantation of ICL with reverse orientation has been reported after different durations. In a case series done by Kaur et al., explantation was done for 11 cases for various reasons [3]. The explantation for reverse orientation was done for 1 out of 11 cases 3 years after implantation. In this case, we reported inverted ICL explantation combined with cataract extraction after 10 years of implantation. Up to our knowledge and search, this is the longest duration reported in the literature. The follow-up for cases of ICL and assessment of vault and orientation are always useful with the help of AS OCT.

AS OCT can determine precisely the ICL vault and orientation. The normal vault values usually range between 250 and 1000 *μ*m [5]. High vault can result from oversizing of the ICL and leads to shallow anterior chamber (AC), high intraocular pressure (IOL), and peripheral anterior synechia (PAS) while low vault which results from undersizing can lead to excessive rotation and anterior subcapsular cataract. In Figure 2, AS OCT is clearly showing the reverse orientation of the ICL with the anterior lens opacity.
